# Mechanical Performance of Flax Fiber Composites with Waste Glass Fibers as a Core Structure

**DOI:** 10.3390/ma15249017

**Published:** 2022-12-16

**Authors:** Anurag Pisupati, Myléne Deléglise Lagardère

**Affiliations:** Center for Materials and Processes, IMT Nord Europe, Institut Mines-Télécom, Université de Lille, 59000 Lille, France

**Keywords:** flax fibers, glass fibers, hybrid composites, fiber waste, mechanical properties

## Abstract

This work sheds light on the first steps towards using glass fiber waste for semi-structural applications. This work aims to improve the properties of random flax fiber composites by incorporating waste glass fibers (WGF) obtained from the fiber production line. The waste glass fibers were incorporated as a core structure between the flax layers to form a hybrid composite. Two routes of manufacturing viz. vacuum infusion and autoclave were used to identify the optimum route to incorporate the WGF in flax fiber composites. The quality of composites was investigated in terms of residual void content and thickness uniformity. Residual void content was identified to be directly proportional to the WGF content in the composites. With the increase in WGF content, the flexural and impact properties were increased by 47% and 117%, respectively, indicating a positive hybridization effect. Furthermore, a global warming potential indicator was identified to be small, indicating the eco-friendliness of these composites.

## 1. Introduction

With the growing implementation of composite materials, it is evident that their end-of-life treatment is an impending concern for manufacturers, especially in the automotive and wind sector. The EU legislation towards composites recycling, Directive 1999/31/EC on the landfill of waste and Directive 2000/76/EC for incineration of waste, and 200/53/EC on the end of life vehicles impose stringent rules on the composite manufacturers. Decarbonizing industry in France is of utmost interest after adopting the national low carbon strategy in 2020 [1]. This new policy set a target of 53 Mt of carbon dioxide emissions in 2030 compared to 84 Mt in 2019. These initiatives call for the implementation and usage of eco-friendly composite materials such as natural fiber composites. However, owing to the limitations in terms of mechanical performance for a few applications, natural fiber composites cannot always be used in industries. Hence, glass fiber composites are almost unavoidable, and furthermore, their existence for the past two decades has caused a rise in composite waste [2].

Composite waste can be mainly categorized into two groups; fiber or prepreg waste from manufacturing and end-of-life composite waste [2]. The re-utilization of the prepreg scrap from the manufacturing line has been well established in the industry [3]. Studies ranging from the manufacturing quality to the mechanical properties of such composites are abundant in literature [4,5,6,7,8,9,10]. It has been proven that the composites manufactured from the prepreg scraps can easily match the conventional composites and can be implemented in several applications [3]. On the other hand, handling composite materials at the end of their life is also of great interest. In the case of thermoplastic-based composites, the composites can be simply shredded and reused for different applications. It should be noted that the recycled composites exhibit lower mechanical properties in comparison with their predecessors [11]. On the other hand, thermoset composites cannot be directly reused in lightweight applications. The recycled aggregates of thermoset composites are known to be used as fillers in concrete applications. However, in the field of composite materials, their re-utilization is not straightforward [12]. Reinforcements from the composites can be obtained either via solvolysis or pyrolysis, currently, both of which may not be economically viable routes for glass fiber reinforcements.

Thomason et al. [13] investigated the effects of the strength degradation of the recovered glass fibers and improved the fiber properties by chemical treatments. Rahimizadeh et al. [14] investigated the mechanical properties of the recycled glass fibers from the wind turbine blades, and they identified that the interfacial shear strength could be recovered by heat treatments. Hasan et al. [15] investigated the use of staple carbon fibers by transforming them into hybrid yarns with a thermoplastic matrix. The mechanical properties of the composites made from those hybrid yarns were a good match to conventional UD carbon composites. Davidson et al. [16] proposed splicing of the fiber waste as a feasible option to produce semi-continuous fiber tows which can be used to manufacture UD composites matching the performance of conventional UD composites.

It can be noticed that several works in the literature discuss the utilization of recycled fibers [17] or agriculture waste [18,19]. Still, the direct incorporation of technical reinforcement waste from fiber manufacturing is rarely addressed. This is either because the waste fibers are reused in fiber preparation by melting again (before sizing) or are considered waste and transferred to landfills. It has been identified that these fibers are a significant contribution to landfills, and a potential application can help reduce this type of waste fibers. Hence, the focus of this study is to understand the reusability of glass fiber waste from the fiber manufacturing line. These waste glass fibers (WGF) can be used in multiple ways and in this work, an effort to use them as a secondary reinforcement in the flax-based composites. Flax fibers were selected owing to their low environmental impact, and higher specific mechanical properties [20,21]. Also, it has been reported that flax/glass hybrid composites exhibit superior mechanical properties [22,23,24]. Hence, in this study, firstly, the morphology of the glass fibers is characterized to understand the physical variation in terms of diameter and length. The surface chemistry of the glass fibers is then understood using the spectral analysis from FTIR. The fibers are then incorporated into composites which are manufactured via compression molding. The quality of composites in terms of thickness, residual voids, and the resultant mechanical properties are discussed.

## 2. Materials and methods

### 2.1. Materials

**Figure 1:**
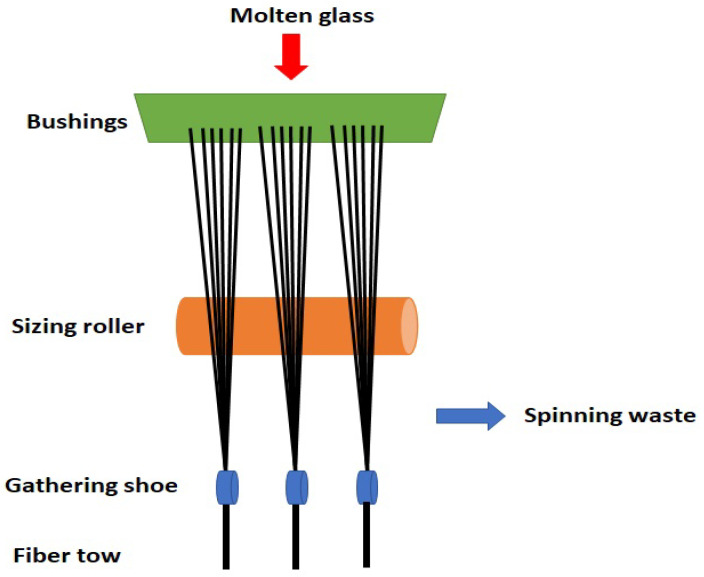
A schematic of glass fiber production line.

**Figure 2:**
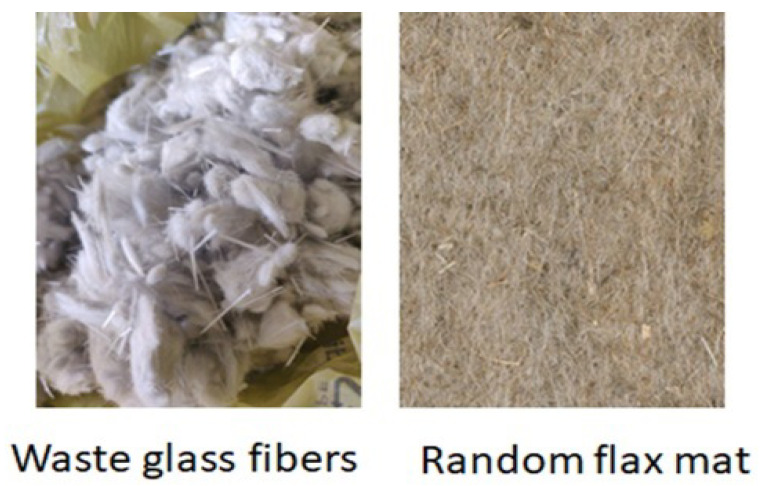
Reinforcements used in this study (**left**) waste glass fibers and random flax mat (**right**).

The waste glass fibers in this work were collected from the production line of glass fibers (see Figure 1). When the molten fibers are extruded from the bushings and passed onto the sizing rollers, the discontinuous fiber bundles stuck to the roller. These fibers are manually collected and are sent to waste fills or recycled. Since these fibers are discontinuous in nature and are difficult to hand, they are usually discarded. In Figure 2, one can notice the discontinuous and agglomerated nature of glass fibers. As a primary reinforcement, a random flax fiber mat with an areal density of 300 g/m${}^{2}$ was used, and a biobased epoxy resin (Greenpoxy 56, Sicomin, France) was used to impregnate the preforms.

### 2.2. Fiber Morphology

To characterize the WGF morphology, the fibers are rubbed over a double-faced adhesive tape. The tape is mounted onto an aluminum specimen holder and transferred into a scanning electron microscope (SEM). A Jeol 6000 SEM (Japan) is used to characterize the fiber morphology. The micrographs are then treated using ImageJ to report the diameter distributions.

### 2.3. FTIR

A ThermoFisher Nicolet S20 Fourier transform infrared spectrometer (FTIR) was used to obtain information on the surface composition of glass fibers. A glass fiber sample of mass 1–2 mg was ground with a Potassium Bromide (KBr) powder and was pelleted using a hand press. The measurements were carried out with a frequency range of 300–4500 cm${}^{-1}$ with a resolution of 2 cm${}^{-1}$. In order to remove the background noise, the same measurements were carried out on pure KBr pellets, and the response was subtracted from the sample spectra.

### 2.4. Composite Manufacturing

**Figure 3:**
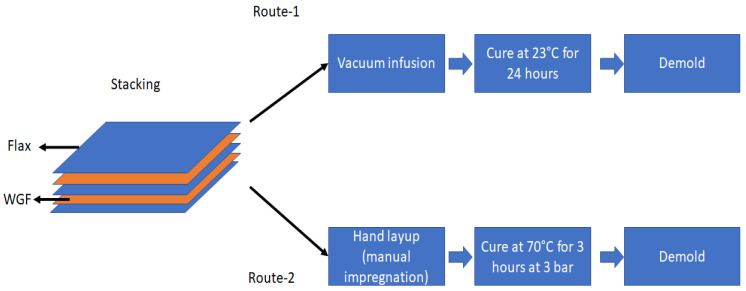
A schematic of manufacturing routes for WGF composites.

In this work, the composites were manufactured using two different techniques viz. vacuum infusion(VI) and autoclave (see Figure 3). All the composites in this work are composed of three layers of random flax fiber mats with varying quantities of glass fiber waste between the layers. The details of the composites are provided in Table 1. The glass fibers are separated and equally distributed manually between the flax layers to achieve the target glass fiber mass fraction ($M_{f,glass}$). The flax fiber mats were not dried before manufacturing. In the case of vacuum infusion, a distribution media was placed over the preform to ensure uniform and faster impregnation times. The VI composites were cured at room temperature for a duration of 24 h and were then transferred to storage. In the case of autoclave manufacturing, similar steps to the VI process were followed, but the preforms were manually impregnated with an epoxy resin to ensure complete impregnation. The impregnated preforms were transferred into an autoclave for consolidation at 70 °C under a consolidation pressure of 3 bars for 3 h. Since the cure duration plays a significant role in the mechanical performance of the composites [25], the cure duration in this study was selected based on the previous study [26]. The composites were then transferred to storage at 23 °C and RH 50% for two days before testing.

### 2.5. Mechanical Tests

Three-point bending tests were conducted on a universal testing machine (Zwick Roell Z010, Germany) according to the standard ISO 14125 to identify the flexural properties of the composites. The samples were of dimensions 75×15 mm${}^{3}$ with a span length of 60 mm. The tests were conducted with a constant crosshead displacement of 1 mm/min. The Charpy impact tests were carried out on a Zwick Charpy impactor. The impact energy was maintained constant for all the tests to report the absorbed energy. The tests were conducted on unnotched specimens. At least six specimens were tested to report the average mechanical properties of composites.

### 2.6. Void Content Measurements

Residual void content within the composites was quantified using the density measurements and calcination process as suggested in [27]. Specimens of dimensions 20×20 mm${}^{2}$ were cut along the central axis of each composite plate. The density of each specimen was measured using the immersion technique with ethanol ($\rho_{eth}=0.798$ g/cm${}^{3}$) as a test liquid. The specimens were then dried for 12 h at 40 °C to remove the presence of residual ethanol and were subjected to calcination in a closed mold at 550 °C. The fiber distribution along the plate was identified by subjecting the specimens from each plate to calcination. This step was carried out for each plate produced to ensure repeatability.

## 3. Results and Discussion

### 3.1. WGF Morphology

**Figure 4:**
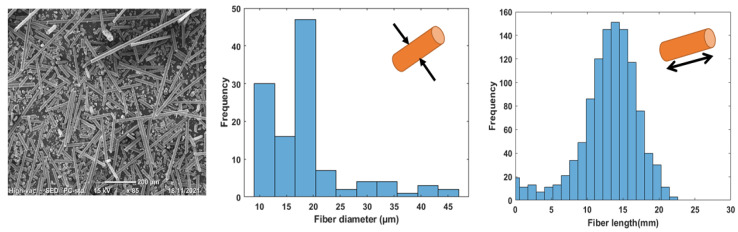
Morphology of WGF: SEM image of fibers (**left**) and fiber diameter distribution (**right**).

WGF fibers are bundled in nature, and it was found to be difficult to unbind and incorporate into composites. Hence even the morphological analysis was carried out on these fiber bundles as shown in Figure 2. The morphological data of WGF is presented in Figure 4. The fiber diameter was about 20 μm on average and exhibited a log-normal distribution. The impurities such as stitch yarns or other fiber grades could have contributed to the larger fiber diameters. The WGF fibers had an average length of 12 mm, however, there were several smaller fibers whose length was between 50–200 μm. During fiber production, these fibers could have been broken when discarded or transported to storage. Furthermore, the smaller fibers will only act as inclusions and not as reinforcing agents. The range of fiber lengths corresponds to the conventional short fiber and long fiber composites [28]. It can be concluded that these fibers can be used as a reinforcement for composite materials.

**Figure 5:**
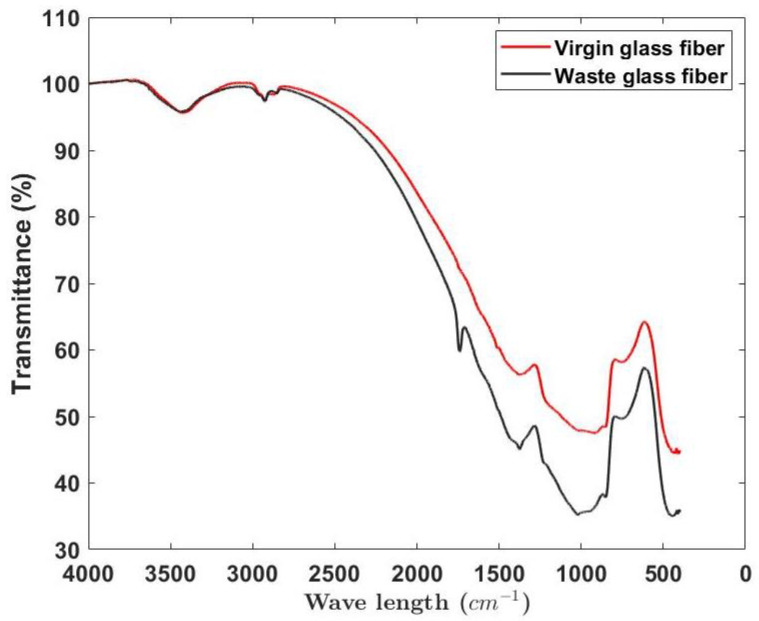
FTIR spectra of virgin and waste glass fibers.

In Figure 5, the nature of the chemical composition of the waste glass fibers can be noticed. To understand it better, a virgin glass fiber sample, i.e., glass fibers that were successfully produced, were compared to the WGFs. The spectrum of virgin glass fiber and WGF in the range of 4000–2500 cm${}^{-1}$ were identical, indicating that both specimens had a similar composition. This range corresponds to the stretching of C-H and NH2-NH bonds. However, below 2500 cm${}^{-1}$, the spectrum of WGF diverges from the virgin fibers. This divergence becomes significant between the range of 1500–500 cm${}^{-1}$. The spectrum within this range indicates the probable presence of silane groups. Since the WGFs did not undergo the complete production steps, the difference in the spectral response can be attributed to the partial absence of the silane groups.

### 3.2. Residual Void Content

**Figure 6:**
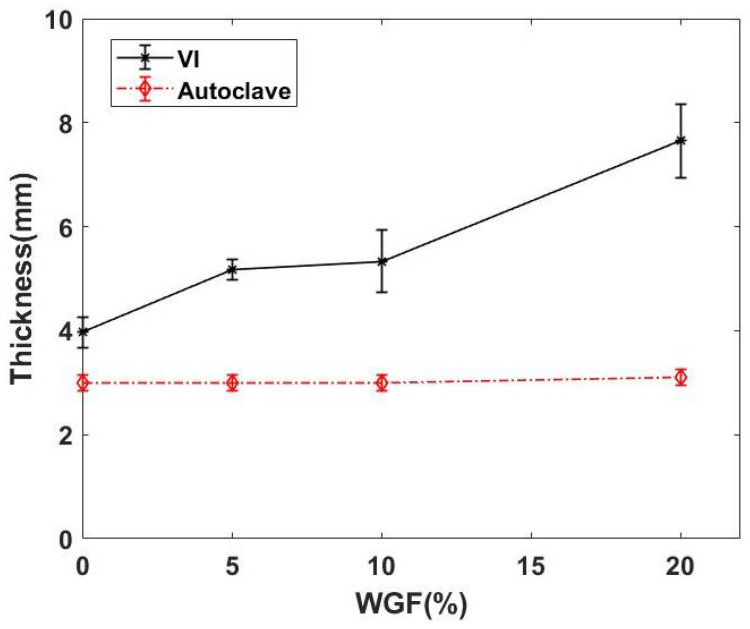
Average thickness of WGF composites manufactured using two different routes.

Figure 6 shows the average thickness of composites manufactured using two different techniques. In the case of VI composites, the thickness increases with an increase in WGF content. This increased WGF content can be attributed to the increased rigidity of the preform, and hence it was difficult to achieve uniform thickness. The thickness of the VI composites increased by about 96% when 20% WGF was added to the flax fiber preforms. Since the compaction pressure was limited to 0.9 bar, the preform could not reach a constant thickness in every case. This increase in thickness can lead to a decrease in the estimated fiber volume fraction and also high residual void content within the composites. However, in the case of the autoclave, owing to higher compaction pressures, a uniform thickness was achieved.

**Figure 7:**
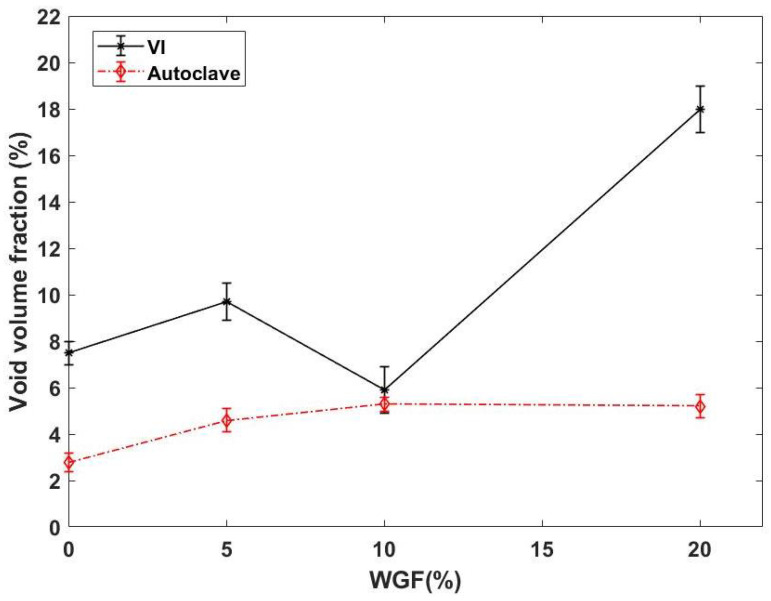
Residual void volume fraction of WGF composites manufactured using two different routes.

The residual void fraction ($V_{\phi }$) of WGF composites is shown in Figure 7. It can be seen that the $V_{\phi }$ is highly dependent on the WGF content similar to the thickness values. In the case of VI composites, $V_{\phi }$ was found to be very high compared to autoclave composites. Owing to the high $V_{\phi }$ in WGF-20 composites, the composites cannot even be considered for semi-structural applications. This high $V_{\phi }$ in VI composites could have been caused by two phenomena. Firstly, the low compaction pressure could have led to the formation of less torturous flow paths through which the resin would have flown faster. Since WGFs were bundled in nature, this faster flow would not have completely impregnated the fiber bundles, thus contributing to increasing in $V_{\phi }$ content. The second reason for this high $V_{\phi }$ can be due to the usage of the distribution medium. By using a distribution medium in a VI process, the longitudinal flow velocity can be significantly increased; however, if the preform has a significant thickness, the through-thickness impregnation can be poor. In the case of WGF-20, these two phenomena could have contributed to high $V_{\phi }$ content.

In the case of autoclave composites, the $V_{\phi }$ identified in this work is lower than the values reported in the literature. Fiore et al. identified a 6.2% of residual voids in the case of flax/glass hybrid composites manufactured by compression molding [29]. Usually, when composites are manufactured using a pre-impregnated stack, the $V_{\phi }$ is dependent on the air evacuation capacity of the preform [30]. However, in this work, the preforms were immediately consolidated to the target thickness without any dwell time under vacuum. This rapid consolidation leads to the closure of the flow paths causing the air bubbles to be entrapped within the preform. This phenomenon could have led to a relatively higher $V_{\phi }$ compared to the other composites manufactured using autoclave [31]. In this study, the $V_{\phi }$ increases from 2.8% to 5.2% with the addition of 10% WGF and then reaches a threshold value. This increase can be attributed to the torturous flow path caused by the random fiber arrangement and the bundled nature of WGF (see Figure 2) leading to the mechanical air entrapment. As the WGF content increases, these impenetrable zones also increase, thus contributing to an increase in residual void volume fraction. The threshold of 5.2% can be explained by the application of vacuum, which ensured that the entrapped air has been evacuated. Similar behavior was also identified by Ramlee et al. in the case of hybrid natural fiber composites [32]. It can thus be concluded that if WGFs are used as secondary reinforcements, a process with high compaction pressures should be adopted to obtain good-quality composites.

### 3.3. Flexural Properties

**Figure 8:**
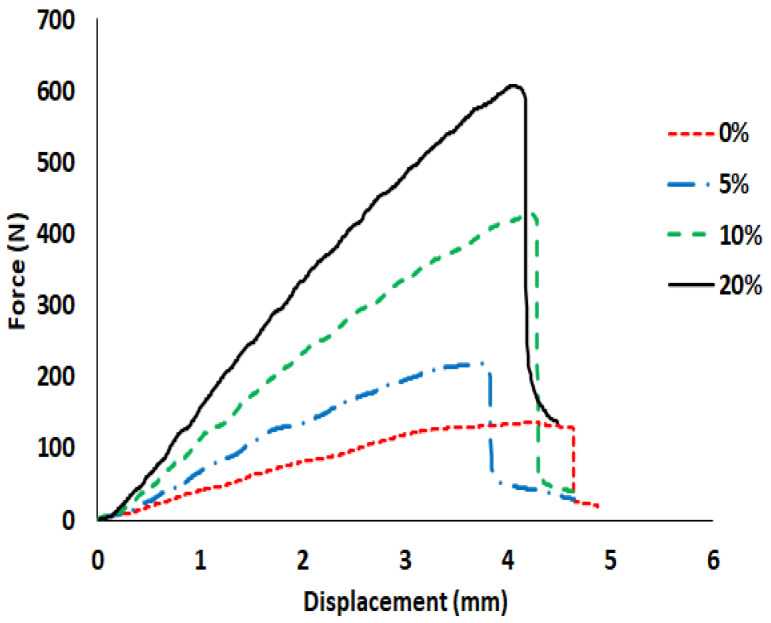
Load-displacement curves of different composites under flexural loading.

**Figure 9:**
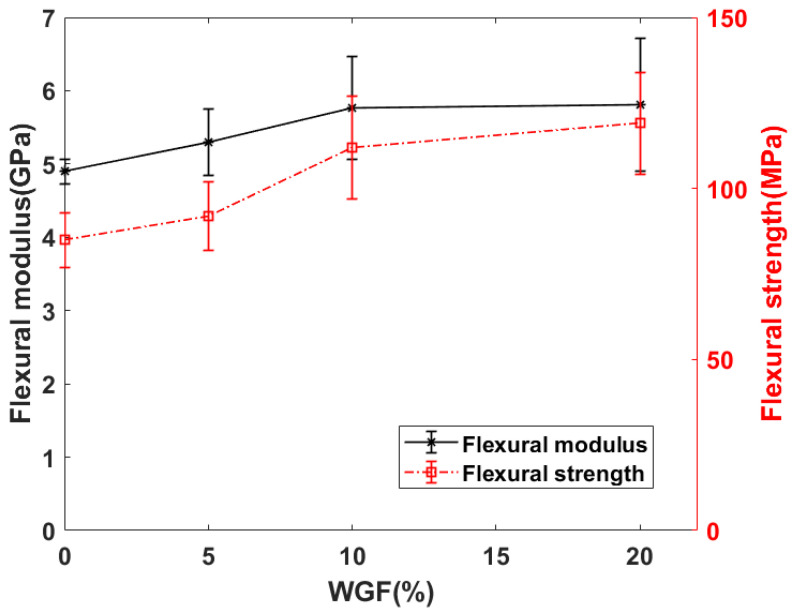
Flexural properties of WGF composites.

The flexural properties of WGF composites manufactured using autoclave are presented in Figure 8 and Figure 9. Since the VI composites exhibited poor quality, their mechanical properties were not investigated. Similar to the residual void content, the flexural properties were identified to be dependent on the WGF content. With the addition of the WGF to flax fibers, a positive hybridization effect can be noticed in the composite’s flexural behavior. The flexural properties tend to reach a threshold value at 10% WGF content in the composites (see Figure 9). This threshold can be owing to the high residual void content within the composites and also can be due to poor stress transfer between the WGF and the matrix.

In Figure 8, the force-displacement curves of the composites indicate that there exists no gradual failure during flexural tests, unlike the woven hybrid composites [33], even after the contribution of two different reinforcements. It can thus be argued that there is a positive hybridization effect, but the failure is sudden without any premonition. Furthermore, the WGF also affected the ductility of the composites. Pure flax composites exhibited a failure strain of 4%, whereas the WGF-20 had a failure strain of 3.6%. This decrease in failure strain also indicates the absence of pseudo-ductility, which many hybrid composites claim to be advantageous [34]. However, in the current case, it cannot be considered disadvantageous. The addition WGF significantly improved the flexural properties, which is a significant contribution that can further be improved by controlling the residual void content within the composites.

It can be argued that considering the large standard deviations, the flexural properties of WGF-5, WGF-10, and WGF-20 are the same. Hence, the flexural properties were subjected to ANOVA analysis to identify the *p*-values. When WGF-5 and WGF-10 were compared, the *p*-value was about 0.01 (<0.05), indicating that values were statistically different. However, when WGF-10 and WGF-20 were compared, the *p*-value was greater than 0.05. This can also be seen in Figure 9, where the average values of the flexural properties were almost similar. It can thus be concluded that an addition of 10% WGF is sufficient to improve the flexural modulus and strength by 18% and 34%, respectively. In Table 2, a comparison has been drawn between the flexural properties of different composites in the literature and the current work. It can be seen that the WGF composites are on par with glass fiber composites in terms of specific strength indicating that the WGF composites can be an alternative to conventional short-fiber composites. The specific modulus can be improved by decreasing the residual void content within the composites.

### 3.4. Impact Resistance

**Figure 10:**
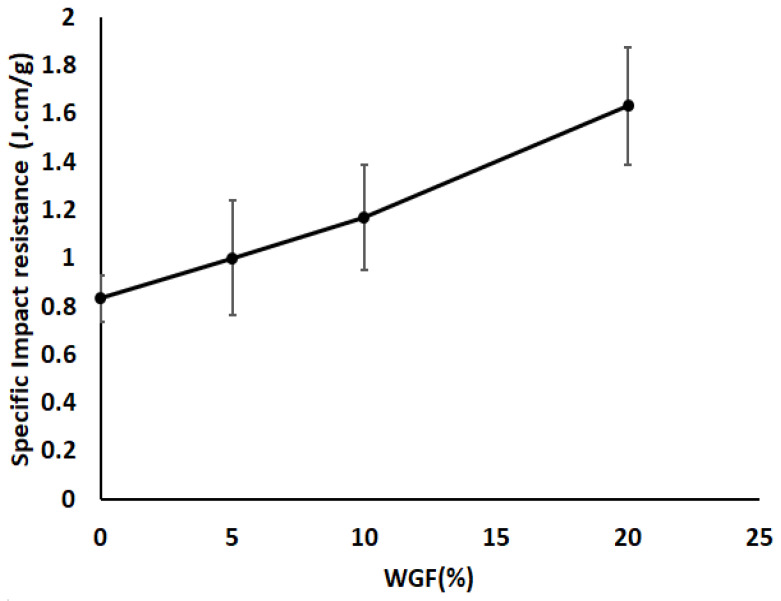
Specific impact resistance of WGF composites.

In Figure 10, the impact performance of the hybrid composites can be noticed. Similar to the flexural properties, the specific impact properties were directly proportional to the WGF content. This increase in the impact resistance can be explained owing to the difficult crack propagation and good energy dissipation within the core structure containing WGF. Since WGF are randomly oriented between the flax non-wovens, an improvement of 114% in impact resistance was observed with an addition of 20% WGF. This improvement is quite significant; however, it should be noted that the weight of the composite has also increased significantly, owing to the incorporation of glass fibers. Monti et al. [41] found that the impact resistance of short glass fiber composites with 20% mass fraction was around 26.4 kJ/m${}^{2}$, indicating that the composites in this work can match the short glass fiber composites. Furthermore, since the composites are made of epoxy, the brittleness can be reduced by choosing better impact-resistant matrix systems. Wambua et al. [42] reported a 25 kJ/m${}^{2}$ impact resistance for natural fiber composites with a 50% fiber mass fraction. In the current study, we surpass this value significantly only by the addition of 10% of WGF.

### 3.5. Environmental Impact

The global warming potential (GWP) indicator obtained from different LCA studies was used to understand the environmental impact of the composites developed within this study [43,44,45,46]. Firstly, using the GWP indicator, the need to use the flax fibers over glass fibers can be established. Vanderwerf et al. [47] reported that to produce a flax fabric, the GWP indicator is 1.36 kgCO${}_{2}$eq whereas for glass fibers the value is in between 1–2 kgCO${}_{2}$eq. The GWP value for flax fibers greatly varies depending on the factors considered during LCA [48,49]. Fernando et al. found that in the case of flax mats or composites, the GWP is actually negative. On the other hand, the energy required to produce one kilogram of epoxy resin is about 137.09 MJ [50], whereas the energy requirements to produce one kilogram of glass fiber and flax fiber is about 48.33 MJ, and 4.4 MJ respectively [20,51]. Since it requires no extra energy for the waste glass fibers, the incorporation of these fibers into composites can greatly decrease the overall cost and also the environmental effect. Also, the waste glass fibers were used along with flax mats, the GWP indicator does not increase as these fibers are bi-products of the actual glass filaments [43,46] ensuring the overall GWP is less than actual glass fiber composites.

## 4. Conclusions

In this work, the incorporation of WGF and its influence on the quality and mechanical performance of composites was presented. The WGFs was identified to be an ideal secondary reinforcement for semi-structural applications where the weight of the composite does not play a significant role in product design. A proper selection of manufacturing processes such as the closed mold process where the consolidation pressures are higher than the OoA process is necessary to produce these composites owing to their poor compressibility. The addition of WGF to flax fiber composites improved the flexural and impact properties. The flexural properties were improved by 47% with the addition of 20% WGF whereas the impact properties were improved by 114%. Finally, the lower GWP values indicate the eco-friendliness of the WGF composites. Further studies on the durability such as hygrothermal aging and fatigue of these hybrid composites will be carried out.

## Figures and Tables

**Table 1:** Composition of composites with WGF.

Designation	$M_{f,\mathrm{glass}}$	$M_{f,\mathrm{flax}}$
WGF-0	0	28
WGF-5	5	28
WGF-10	10	28
WGF-20	20	28

**Table 2:** Comparison of specific flexural properties with literature.

Flexural Strength	Flexural Modulus	Density	Specific Strength	Specific Modulus	Reinforcement	Ref
(MPa)	(GPa)	(g/cm${}^{3}$)	(MPa·cm${}^{3}$/g)	(GPa·cm${}^{3}$/g)		
119.8	5.1	1.16	103.27	4.39	glass	[35]
115.3	8.1	1.26	91.5	6.42	flax	[36]
97.5	7.3	1.27	76.77	5.47	flax	[37]
120.8	8.3	1.31	92.4	6.33	glass	[37]
50	3.9	1.29	38.76	3.02	kenaf	[38]
90	6.4	1.3	69.23	4.92	hemp	[39]
41.8	NA	1.12	37.32	NA	glass	[40]
92.0	5.3	1.3	73.35	4.23	This work	
112.0	5.8	1.3	87.28	4.50	This work	
119.0	5.8	1.4	87.84	4.29	This work	

## Data Availability

All the data is presented in this manuscript. Supplementary information can be gathered from the authors.
